# Incorporating foot assessment in the comprehensive geriatric assessment

**DOI:** 10.1186/s12877-021-02164-3

**Published:** 2021-04-01

**Authors:** Rebecca K. Iseli, Gregory Duncan, Elton K. Lee, Ellen Lewis, Andrea B. Maier

**Affiliations:** 1 Department of Medicine and Aged Care, @AgeMelbourne, The Royal Melbourne Hospital, The University of Melbourne, Melbourne, Victoria Australia; 2 Faculty of Pharmacy and Pharmaceutical Sciences, Monash University, Parkville, Australia; 3 Eastern Health Clinical School, Monash University|, Box Hill, Victoria Australia; 4 Podiatry Department, The Royal Melbourne Hospital, Parkville, Victoria Australia; 5 Department of Human Movement Sciences, @AgeAmsterdam, Vrije Universiteit Amsterdam, Amsterdam Movement Sciences, Amsterdam, The Netherlands; 6 Healthy Longevity Translational Research Program, Yong Loo Lin School of Medicine, National University of Singapore, Singapore; Centre for Healthy Longevity, National University Health System, Singapore, Singapore

**Keywords:** Foot, Foot diseases, Aged, Geriatric assessment

## Abstract

**Background:**

Foot problems are common in older adults and associated with poorer physical function, falls, frailty and reduced quality of life. Comprehensive Geriatric Assessment (CGA), a multidisciplinary process that is considered the gold standard of care for older adults, does not routinely include podiatry assessment and intervention in hospitalized older adults.

**Aims:**

To introduce foot assessment to inpatient CGA to determine prevalence of foot disease, foot disease risk factors and inappropriate footwear use, assess inter-rater reliability of foot assessments, determine current podiatry input and examine associations between patient characteristics and foot disease risks.

**Methods:**

Prospective, observational cohort study of older adults on geriatric rehabilitation wards. Foot assessment completed using the Queensland Foot Disease Form (QFDF) in addition to routine CGA.

**Results:**

Fifty-two patients (median age [inter-quartile range] 86.4 [79.2–90.3] years, 54% female) were included. Six patients (12%) had foot disease and 13 (25%) had a ‘high risk’ or ‘at risk’ foot. Foot disease risk factor prevalence was peripheral arterial disease 9 (17%); neuropathy 10 (19%) and foot deformity 11 (22%). Forty-one patients (85%) wore inappropriate footwear. Inter-rater agreement was substantial on presence of foot disease and arterial disease, fair to moderate on foot deformity and fair on neuropathy and inappropriate footwear. Eight patients (15%) saw a podiatrist during admission: 5 with foot disease, 1 ‘at risk’ and 2 ‘low risk’ for foot disease. Patients with an at risk foot or foot disease had significantly longer median length of hospital stay (25 [13.7–32.1] vs 15.2 [8–22.1] days, *p* = 0.01) and higher median Malnutrition Screening Test scores (2 [0–3] vs 0 [0–2], *p* = 0.03) than the low-risk group. Patients with foot disease were most likely to see a podiatrist (*p* < 0.001).

**Conclusion:**

Foot disease, foot disease risk factors and inappropriate footwear are common in hospitalized older adults, however podiatry assessment and intervention is mostly limited to patients with foot disease. Addition of routine podiatry assessment to the multidisciplinary CGA team should be considered. Examination for arterial disease and risk of malnutrition may be useful to identify at risk patients for podiatry review.

**Supplementary Information:**

The online version contains supplementary material available at 10.1186/s12877-021-02164-3.

## Introduction

Changes to the feet occur with age, leading to high prevalence of foot problems such as foot pain, deformity, muscle weakness and reduced range of motion [1, 2]. This contributes to morbidity in older populations: foot problems have been associated with a higher risk of falls [3], reduced quality of life [4–7], frailty [8] and lower independence in activities of daily living (ADLs) [9, 10]. Lower independence in ADLs is associated with nursing home admission [11–13]. Poor foot care may also be an indicator of self-neglect [14, 15]. Inappropriate footwear is associated with impaired balance with associated risk of falling [16] and foot disease [17]. Foot disease, defined as foot ulceration, infection, critical ischaemia and Charcot neuroarthropathy [18], represents more severe foot pathology. Foot disease is associated with many chronic illnesses and increases in prevalence with age [2, 19], affecting approximately 10% of hospital inpatients [1, 20]. Foot disease usually occurs in people with risk factors such as foot deformity, peripheral arterial disease, peripheral neuropathy, previous ulceration and/or previous amputation [21]. In community dwelling older adults the presence of foot disease is associated with poorer physical function [22].

Despite the impact of foot problems and foot disease on older adults, the foot is frequently neglected by both patients and healthcare providers [23, 24]. The gold standard of care for older adults is the Comprehensive Geriatric Assessment (CGA), a multidimensional, multidisciplinary assessment of medical, social, psychological and functional needs that leads to the development of a coordinated care plan [25]. Podiatry interventional studies have shown reduced risk of falls in community dwelling older adults [26], and integration of a podiatry assessment in the CGA is recommended as part of falls assessment and prevention programs [27]. Inpatient CGA programs, on the other hand, do not routinely include podiatry assessment [28, 29].

The aim of this study was to introduce a standardised foot assessment to older adults undergoing inpatient CGA, to: (1) determine the prevalence of foot disease, foot disease risk factors and inappropriate footwear use; (2) assess the inter-rater reliability of foot assessments; (3) determine the frequency of podiatry assessment; and (4) evaluate associations between patient characteristics and foot disease and foot disease risk factors.

## Methods

### Study design

#### Population

This study is embedded in REStORing health of acutely unwell adulTs (RESORT), an ongoing prospective, observational, longitudinal inception cohort investigating the characteristics and outcomes of geriatric rehabilitation patients at the Royal Melbourne Hospital (Melbourne, Victoria, Australia). Patients included in the RESORT study are assessed within 48 h of admission and discharge with a multidisciplinary, multi-dimensional CGA. Recruitment for wave 1 of RESORT commenced on October 15, 2017. Each annual RESORT wave recruits approximately 600 patients. This sub study, a pilot study adding foot assessment during the geriatric rehabilitation admission, recruited a convenience sample of inpatients from wave 3 between February 12 and March 19, 2020. A power calculation of sample size for foot disease prevalence was 138 patients (based on estimated prevalence of 10%, Z statistic of 1.96 and allowable error of 0.05) [30]. This study was planned to run for 3 months, with an expected sample size of approximately 200 patients, however due to the COVID-19 pandemic recruitment ceased early. Patients were excluded from this study if they were receiving palliative care at admission, transferred to acute care prior to consenting to the study, unable to provide informed consent with no nominated proxy or were discharged prior to foot assessment. The study was performed in accordance with the Declaration of Helsinki and approved by the Melbourne Health Human Research and Ethics Committee (HREC) (approval number 2017.085).

#### Patient characteristics

Patient demographics, reason for admission and history of falls were collected from medical records and patient surveys. Physical function was assessed with the Functional Ambulation Classification [31], a scale from 0 to 5 recording ambulation ability, and the Short Physical Performance Battery (SPPB) [32], a three component assessment, scored from 0 to 12 points, of balance, 4-m walk and a chair stand test. Functional status was assessed using Katz Activities of Daily Living [33], a 6 item scale of independence in personal ADLs, and Instrumental Activities of Daily Living [34], a measure of independence in domestic and community ADLs, scored from 0 to 8. For the ADL and mobility assessments higher scores indicate greater independence and better physical function, respectively. Nutritional status was assessed with the Malnutrition Screening Tool (MST): a score of 2 or more indicates risk of malnutrition [35]. Pressure injury risk assessment was undertaken using the Braden Scale, which evaluates six domains to determine risk score: scores below 18 suggest increased risk of pressure injury [36]. Medical co-morbidity was assessed with the Cumulative Illness Rating Scale (CIRS) [37], a measure that rates disease burden across major organ groups (higher scores indicating greater severity). Frailty was assessed using the Clinical Frailty Scale, a 7-point scale, with higher scores indicating greater frailty [38]. Discharge destination (community or residential aged care facility) and death during geriatric rehabilitation admission were recorded from the medical history.

#### Foot risk factors and foot disease assessment

Assessment for foot disease and foot disease risk factors was undertaken by trained clinical researchers using the Queensland Foot Disease Form (QFDF), a 57 item, validated assessment instrument [18] (Additional file 1: Appendix I). The QFDF collects data on demographics, medical co-morbidities, foot disease risk factors and foot disease. Foot disease is defined as foot ulceration, infection, critical arterial disease (toe pressure of < 30 mmHg or Ankle Brachial Index < 0.5) and/or Charcot neuroarthropathy. Foot disease risk factors are deformity (defined as a score of 3 or more on a 6-point scale of deformity), peripheral neuropathy, peripheral arterial disease (toe pressure < 70 mmHg), previous ulceration and previous amputation. Footwear most commonly used indoors and outdoors was classified as appropriate or inappropriate. Inappropriate footwear was defined as footwear that does not have the following components: low heel, non-slip sole, supported heel collar or fastening mechanism [39]. The QFDF assigns a level of foot risk based on presence of foot disease risk factors: low (no neuropathy or PAD), at risk (neuropathy or PAD), high risk (foot deformity with neuropathy and/or PAD; previous foot disease) or acute foot disease.

Standardised training in use of the QFDF was undertaken with the assistance of a podiatrist (EL). A sample of foot assessments was repeated by the second assessor, blinded to the initial assessment, to check inter-rater reliability. The patient’s treating team was notified of the outcome of the foot assessment where previously unidentified foot disease was noted, and recommendation for podiatry referral was made where appropriate. A number of patients also underwent foot assessment by a podiatrist as part of their usual care; this was recorded with the hospital podiatry assessment tool (Additional file 1: Appendix II). Referrals to podiatry were made by clinical staff as needed, usually by nursing staff based on skin assessments. The hospital podiatry assessment tool includes similar data to the QFDF; however, diagnosis of foot deformity and type of footwear are recorded by the podiatrist descriptively rather than using a prescribed format as with the QFDF.

Inter-rater reliability was assessed using percentage agreement overall and Cohen’s Kappa co-efficient was calculated for 5 patients comparing researcher assessments and 8 patients comparing researcher to hospital podiatrist assessment. Agreement based on Kappa values was defined as: > 0.8 excellent; 0.61–0.80 substantial; 0.41–0.6 moderate and < 0.40 fair to poor [40, 41].

Assessments were captured on paper forms and then de-identified and managed using the Research Electronic Data Capture (REDCap) [42] tool. Data was exported to SPSS for Windows version 26 (IBM Corp. Released 2016. IBM SPSS Statistics for Windows, Version 24.0. Armonk, NY: IBM Corp) for analysis.

### Statistical analysis

Descriptive statistics were used to summarise the patients’ baseline characteristics. Data with a normal distribution is presented as mean and standard deviation; data with a non-normal distribution is presented as median and interquartile range; and categorical variables are presented as proportions. The baseline characteristics of the foot risk groups were compared using the Kruskal-Wallis H test (for more than 2 groups) or Mann-Whitney U test (for 2 groups) for nonparametric continuous and ordinal data and Fisher’s exact test for categorical data. To reduce type 1 error, the level of significance for all analyses was set at *p* < 0.05.

## Results

The baseline characteristics of the 52 included patients are given in Table 1: median age was 86.4 [IQR 79.2–90.3] years, 54% were female. The median values for admission Katz ADL, iADL, SPPB, FAC and CFS demonstrate low independence, impaired mobility and moderate frailty in the cohort. A majority of patients (69%) had fallen in the last year. Diabetes mellitus was diagnosed in 15 (29%) patients.

**Table 1:** Characteristics of included geriatric rehabilitation inpatients at admission

Characteristic	***N*** = 52
Age, years	86.4 [79.2–90.3]
Female, *n* (%)	28 (54)
Falls in last 12 months, *n* (%)	35 (69) (*n* = 51)
Number of falls in past year	2 [1–3.5]
Functional Ambulation Classification	3 [0.8–3] (*n* = 50)
Standardised Physical Performance Battery	2 [0–5] (*n* = 50)
Katz ADL	2 [1–3]
Instrumental ADL	1 [1–2.8]
Malnutrition Screening Tool	1 [0–2] (*n* = 51)
Braden score	18 [16–20]
Cumulative Illness Rating Scale	1.9 [1.5–2.3]
Clinical Frailty Scale	6 [4–7] (*n* = 47)

Data are presented as median [IQR] unless otherwise stated

*ADL* Activities of Daily Living

### Foot disease, foot disease risk factors and inappropriate footwear

Six patients (12%) had foot disease (pressure injury (4/6), neuroischaemia (1/6) and trauma (1/6)). The prevalence of foot disease risk factors was: mild to moderate peripheral arterial disease 9 (17%), neuropathy 10 (19%) and foot deformity 11 (22%). Reported footwear was inappropriate in 41 patients (85%, *n* = 48): the most commonly worn footwear indoors and outdoors was slippers (34%) and moccasins (33%) respectively. Thirteen (25%) patients were assessed as having an ‘at risk’ or ‘high risk’ foot. Eight patients (15%) saw a podiatrist during their hospital admission: 5 had foot disease, 1 was at risk and 2 were low risk of foot disease. All patients that were seen by a podiatrist required an intervention, which included advice, nail care, debridement and wound dressings.

### Patient characteristics with and without foot disease

Differences between patients based on risk foot or foot disease are summarised in Table 2. Patients with foot disease were significantly more likely to have podiatry assessment during hospitalisation. There was a trend towards an association between age and foot risk (*p* = 0.08). When risk category was dichotomised (low risk patients versus at risk, high risk and foot disease patients), patients with higher foot risk or foot disease had significantly longer median length of hospital stay (25 [13.7–32.1] vs 15.2 [8–22.1] days, *p* = 0.01) and higher median MST scores (2 [0–3] vs 0 [0–2], *p* = 0.03) than the low-risk group.

**Table 2:** Patient characteristics stratified by foot disease risk

	Low risk (*n* = 33)	At risk (*n* = 6)	High risk (*n* = 7)	Foot Disease (*n* = 6)	*P* value
Age, years (median [IQR])	84.5 [78.5–89.5]	80.1 [63.9–89.9]	90 [87.4–92.1]	89.1 [85.7–93.1]	0.08^#^
Female, n (%)	20 (61)	2 (33)	3 (43)	3 (50)	0.59^
Falls in last 12 months, n (%)	23 (70)	2 (40) (*n* = 5)	5 (71)	6 (100)	0.10^
Deformity, *n* (%)	4 (12)	0 (0)	6 (86)	1 (20) (*n* = 5)	0.001^
Neuropathy, *n* (%)	0 (0)	4 (67)	5 (71)	1 (16.7)	< 0.001^
Mild or moderate PAD, *n* (%)	0 (0)	4 (67)	2 (29)	3 (50)	< 0.001^
Inappropriate footwear, *n* (%)	26 (90) (*n* = 29)	5 (83)	5 (71)	5 (83.3)	0.52^
Podiatry assessment, *n* (%)	2 (6)	1 (17)	0 (0)	5 (83)	< 0.001^
FAC admission (median, [IQR])	3 [2–3] (*n* = 27)	2 [0–3.3]	1 [0–3]	2 [0–3.3]	0.84^#^
SPPB (median, [IQR])	3 [0–5] (*n* = 32)	0 [0–6] (*n* = 5)	0 [0–5]	1 [0–5.3]	0.79^#^
Katz ADL (median, [IQR])	2 [1–4]	1.5 [0.8–3.8]	1 [1–3]	2 [0.8–2.5]	0.31^#^
iADL (median, [IQR])	1 [1–2]	2 [0–3.3]	1 [1–3]	1[0–3]	0.12^#^
MST (median, [IQR])	0 [0–2]	1.5 [0–2.3]	2 [2–3]	1 [0–3] (*n* = 5)	0.11^#^
Braden scale (median, [IQR])	18 [17–20]	16 [15.5–20.3]	17 [15–19]	18 [17.5–18]	0.48^#^
CIRS (median, [IQR])	1.8 [1.5–2.4]	2.3 [1.7–2.4]	1.9 [1.7–2]	1.8 [1.3–2]	0.56^#^
CFS (median, [IQR])	6 [4–7] (*n* = 31)	6.5 [4.5–7] (*n* = 4)	6 [5–6.3] (*n* = 6)	6.5 [5–7]	0.42^#^
Length of stay, geriatric rehabilitation, days (median, [IQR])	15.2 [8–22.1]	27.1 [12.7–37.7]	25.8 [12.5–32.8]	24.5 [14.5–27.7]	0.1^#^
Discharged home, *n* (%)	23 (70)	4 (67)	4 (57)	2 (33)	0.41^
Mortality, *n* (%)	2 (15)	0 (0)	0 (0)	1 (17)	0.59^

Group comparisons: ^Fisher’s exact test, ^#^Kruskal-Wallis H test

Abbreviations: *ADL* Activities of Daily Living, *BMI* Body Mass Index, *CFS* Clinical Frailty Scale, *CIRS* Cumulative Illness Rating Scale, *FAC* Functional Ambulation Classification, *iADL* Instrumental Activities of Daily Living, *IQR* Interquartile range, *MST* Malnutrition Screening Tool, *PAD* Peripheral Arterial Disease, *SPPB* Standardised Physical Performance Battery

### Inter-rater reliability

There was substantial to excellent agreement on presence of foot disease (Cohen’s kappa 0.75–1; percentage agreement 92%), moderate to substantial agreement on presence of arterial disease (Cohen’s kappa 0.5–1; percentage agreement 92%), fair to moderate agreement on foot deformity (Cohen’s kappa 0.33–0.5, percentage agreement 63%) and fair agreement on neuropathy (Cohen’s kappa 0–0.3, percentage agreement 63%) and inappropriate footwear (Cohen’s kappa 0–0.2, percentage agreement 67%).

## Discussion

Foot disease risk factors and foot disease were common in this cohort. Podiatry assessment and intervention was undertaken for most patients with foot disease but for few patients with an at risk or low risk foot. Inter-rater reliability testing confirmed that there was substantial agreement on presence of foot disease and arterial disease, however agreement was poorer for neuropathy, deformity and inappropriate footwear. Presence of peripheral arterial disease may therefore be a useful indicator for podiatry referral for patients with an at risk foot. Inter-rater reliability for footwear and neuropathy may have been impacted by high rates of cognitive impairment in older adults on sub-acute wards [43]. Differences in training and clinical experience, the small sample size and the use of different assessment forms by researchers and hospital podiatrists may also have contributed to these findings. Nevertheless, the poor inter-rater reliability for these risk factors highlights the difficulty for non-podiatrist clinicians identifying at risk patients.

Risk of malnutrition was higher and length of stay on the Geriatric Rehabilitation ward was longer for patients with foot disease, at risk and high risk feet. There was no difference in Cumulative Illness Rating Scale or Clinical Frailty Scale between the groups, suggesting that this is not due to overall ill-health. The difference in length of stay may be explained by differences in nutritional status, as malnutrition is associated with longer length of hospital stay [44]. Malnutrition is a risk factor for pressure injuries, and nutritional interventions are recommended in prevention and treatment of pressure injuries [45]. Although two thirds of cases of foot disease in this cohort were pressure injuries, there was no difference in Braden scale between the foot disease group and other groups. A previous study also noted that the Braden scale under-estimates risk of foot pressure injury [46]. Risk of malnutrition may be useful to identify at risk patients for podiatry assessment. Although previous studies have shown associations between foot problems (particularly foot pain) [2, 47] and foot disease [48] and poorer physical function, this study did not identify any differences in physical function based on foot disease or foot disease risk factors.

The rate of foot disease in this cohort is similar to other studies in sub-acute populations in Scotland and Australia that reported prevalence rates of 15% [17] and 12% [20] respectively. Diabetes-related foot disease is well recognised and improved outcomes have been achieved in recent years with interdisciplinary care [49], however in this cohort a minority of patients with foot disease and foot disease risk factors were diagnosed with diabetes. This is consistent with other studies of older adults [1, 17, 50]. Numerous other chronic medical conditions are associated with foot disease, such as osteoarthritis [51], chronic renal failure [52], gout [53] and rheumatoid arthritis [54]. Changes to the foot in addition to increased prevalence of chronic medical conditions with age [2, 55] are the likely cause of high rates of non-diabetes related foot disease in this population.

Use of inappropriate footwear was very common in this cohort and similar rates have been reported by other inpatient and outpatient studies of older adults [17, 56, 57]. Footwear interventions have been shown to improve foot pain and function [58, 59]. Multifaceted podiatry intervention, including footwear advice and provision of orthotics, has been shown to reduce the risk of falls, however footwear advice alone has not [26]. Improving footwear is recognised to be a difficult task: low adherence rates for recommended footwear have been reported, mostly due to the cost and aesthetics of appropriate footwear [60].

Given the high prevalence of foot disease, foot disease risk factors and inappropriate footwear, as well as evidence for reduced falls [26] and foot pain [61, 62] from outpatient podiatry intervention in older adults, routine podiatry assessment and intervention should be considered as part of the CGA in geriatric rehabilitation inpatients. An alternative approach to increase the rate of podiatry assessment and intervention in this population, based on this study’s findings, would be to include peripheral arterial disease and risk of malnutrition as indicators for podiatry referral.

### Limitations

The pragmatic approach of comparing researcher foot assessments using the QFDF with podiatry assessments using the hospital assessment tool may have affected inter-rater reliability measures due to differences in documentation of foot deformity and footwear. Use of the QFDF for foot assessments may have underestimated the prevalence of foot problems, given that single foot deformities and integumentary problems are not captured with this assessment. The study was conducted in late summer, which may have impacted the prevalence of foot disease: studies of diabetes related foot disease have found higher rates of infection and amputation in warmer months, whilst amputations in people without diabetes are most common in winter [63, 64]. The study sample size is also a limitation, particularly for examining associations between foot disease, foot disease risk factors and patient characteristics.

## Conclusion

Foot disease, foot disease risk factors and inappropriate footwear are common in older adults admitted to geriatric rehabilitation wards although podiatry assessment and intervention is mostly limited to patients with foot disease. Assessment for peripheral arterial disease and risk of malnutrition may help to identify at risk patients for podiatry input. Interventional studies incorporating podiatrist assessment and intervention into CGA are needed.

## Supplementary Information

**Additional file 1: Appendix I.** The Queensland Foot Disease Form [18]. **Appendix II.** Hospital specific podiatry assessment form (2 pages).

## Data Availability

All data generated or analysed during this study are included in this published article.
