# Supplementary material for: Incorporating foot assessment in the comprehensive geriatric assessment
Source: BMC Geriatr. 2021 Apr 1;21:223. doi: 10.1186/s12877-021-02164-3 (PMC8015740; doi:10.1186/s12877-021-02164-3)
Supplement: Supplementary file 1 — Additional file 1: Appendix I. The Queensland Foot Disease Form [18]. Appendix II. Hospital specific podiatry assessment form (2 pages). [file 12877_2021_2164_MOESM1_ESM.docx]

**Incorporating foot assessment in the Comprehensive Geriatric Assessment**

Rebecca K Iseli^1,2^; Gregory Duncan^3^; Elton K Lee^1^; Ellen Lewis^4^; Andrea B Maier^1,5,6^

**Affiliations:**

*^1^ Department of Medicine and Aged Care, @AgeMelbourne, The Royal Melbourne Hospital, The University of Melbourne, Melbourne, Victoria, Australia*

*^2^ Faculty of Pharmacy and Pharmaceutical Sciences, Monash University, Parkville, Australia*

*^3^ Eastern Health Clinical School, Monash University, Box Hill, Victoria, Australia*

*^4^ Podiatry Department, The Royal Melbourne Hospital, Parkville, Victoria, Australia*

*^5^ Department of Human Movement Sciences, @AgeAmsterdam, Vrije Universiteit Amsterdam, Amsterdam Movement Sciences, Amsterdam, The Netherlands*

*^6^ Healthy Longevity Translational Research Program, Yong Loo Lin School of Medicine, National University of Singapore, Singapore; Centre for Healthy Longevity, National University Health System, Singapore*

**Corresponding author:**

Dr Rebecca Iseli

Department of Medicine and Aged Care, @AgeMelbourne, The Royal Melbourne Hospital, The University of Melbourne, Parkville, Victoria, Australia

ORCiD: 0000-0003-2403-5104

Email: [rebecca.iseli@mh.org.au](mailto:rebecca.iseli@mh.org.au)

**Appendix I: the Queensland Foot Disease Form(18)
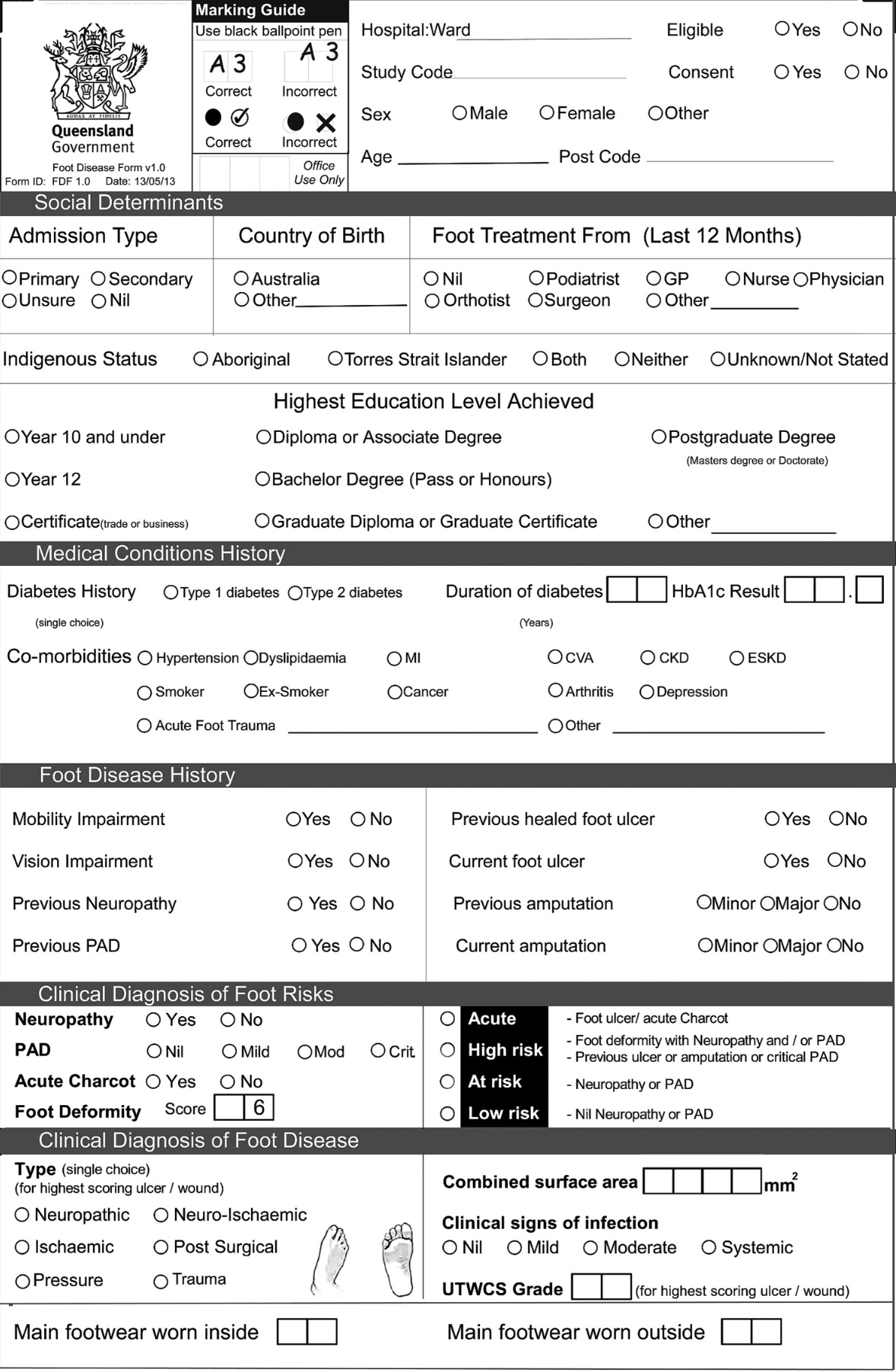
**

**Appendix II: Hospital specific podiatry assessment form (2 pages)
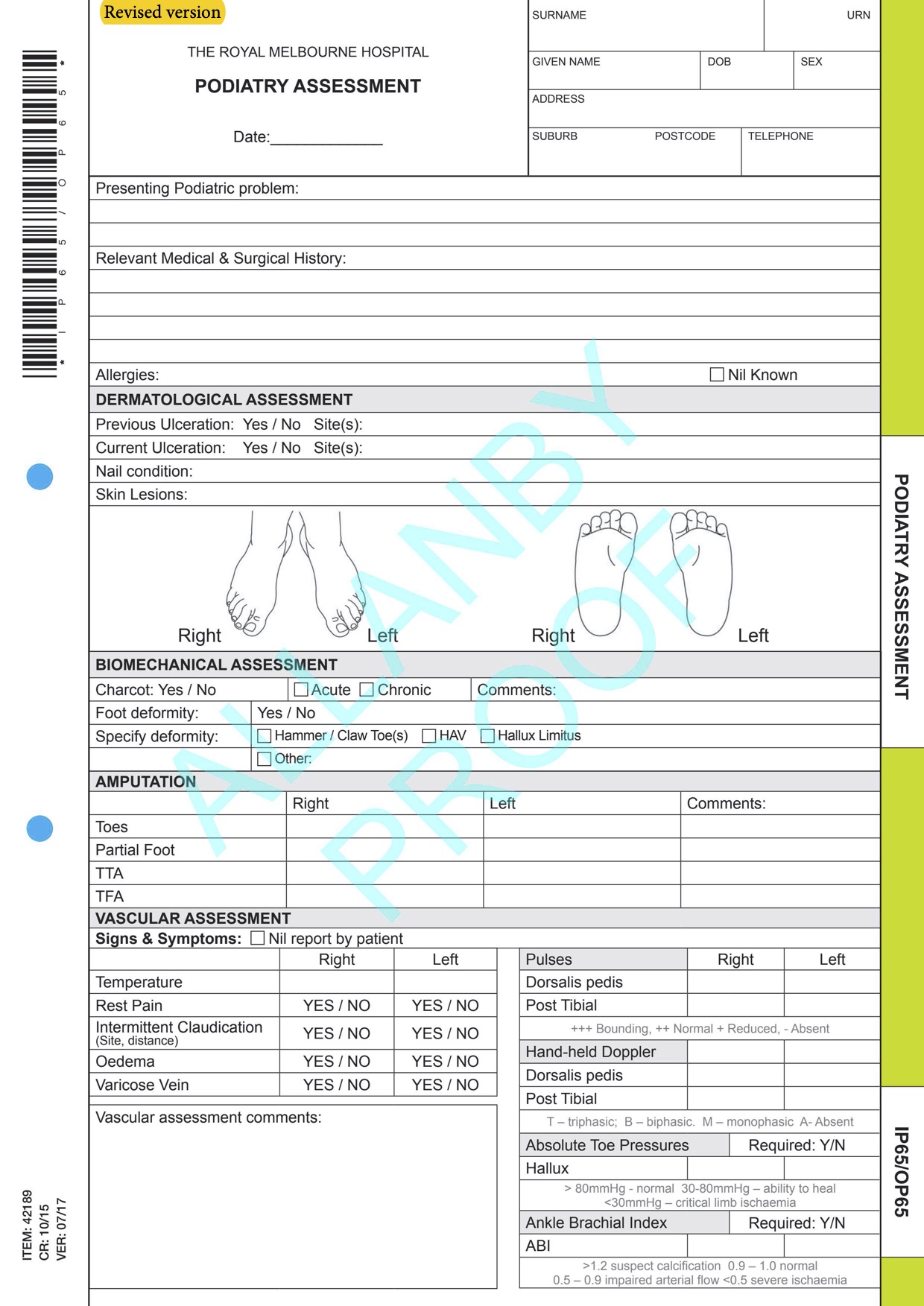
**

**
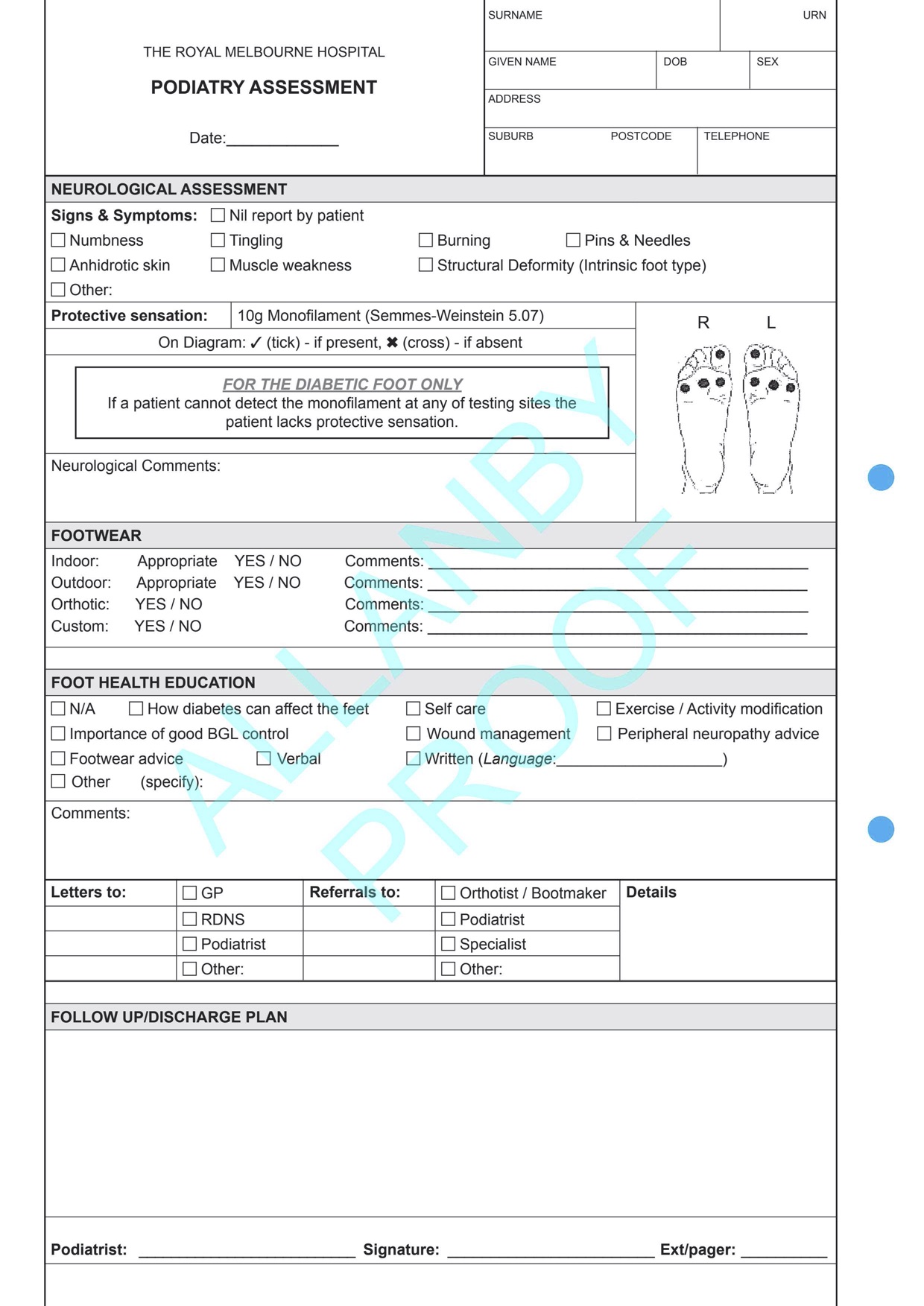
**
